# High level of molecular and phenotypic biodiversity in *Jatropha curcas* from Central America compared to Africa, Asia and South America

**DOI:** 10.1186/1471-2229-14-77

**Published:** 2014-03-25

**Authors:** Luis Rodolfo Montes Osorio, Andres Fransisco Torres Salvador, Raymond Elmar Etienne Jongschaap, Cesar Augusto Azurdia Perez, Julio Ernesto Berduo Sandoval, Luisa Miguel Trindade, Richard Gerardus Franciscus Visser, Eibertus Nicolaas van Loo

**Affiliations:** 1 Plant Breeding, Wageningen University and Research Centre, PO Box 386, 6700 AJ Wageningen, The Netherlands; 2 Agrosystems Research, Plant Research International, Wageningen University and Research Centre, PO Box 616, 6708 AP Wageningen, The Netherlands; 3 Facultad de Agronomía, FAUSAC, Universidad de San Carlos de Guatemala, Guatemala City, Guatemala; 4 Biocombustibles de Guatemala S.A., Guatemala City, Guatemala

**Keywords:** *Jatropha curcas*, Genetic diversity, Phenotypic variation, AFLP, SSR, TRAP, Fatty acid composition, Heritability, RGR, SLA, NAR

## Abstract

**Background:**

The main bottleneck to elevate jatropha (*Jatropha curcas* L.) from a wild species to a profitable biodiesel crop is the low genetic and phenotypic variation found in different regions of the world, hampering efficient plant breeding for productivity traits. In this study, 182 accessions from Asia (91), Africa (35), South America (9) and Central America (47) were evaluated at genetic and phenotypic level to find genetic variation and important traits for oilseed production.

**Results:**

Genetic variation was assessed with SSR (Simple Sequence Repeat), TRAP (Target Region Amplification Polymorphism) and AFLP (Amplified fragment length polymorphism) techniques. Phenotypic variation included seed morphological characteristics, seed oil content and fatty acid composition and early growth traits. Jaccard’s similarity and cluster analysis by UPGM (Unweighted Paired Group Method) with arithmetic mean and PCA (Principle Component Analysis) indicated higher variability in Central American accessions compared to Asian, African and South American accessions. Polymorphism Information Content (PIC) values ranged from 0 to 0.65. In the set of Central American accessions. PIC values were higher than in other regions. Accessions from the Central American population contain alleles that were not found in the accessions from other populations. Analysis of Molecular Variance (AMOVA; P < 0.0001) indicated high genetic variation within regions (81.7%) and low variation across regions (18.3%). A high level of genetic variation was found on early growth traits and on components of the relative growth rate (specific leaf area, leaf weight, leaf weight ratio and net assimilation rate) as indicated by significant differences between accessions and by the high heritability values (50–88%). The fatty acid composition of jatropha oil significantly differed (P < 0.05) between regions.

**Conclusions:**

The pool of Central American accessions showed very large genetic variation as assessed by DNA-marker variation compared to accessions from other regions. Central American accessions also showed the highest phenotypic variation and should be considered as the most important source for plant breeding. Some variation in early growth traits was found within a group of accessions from Asia and Africa, while these accessions did not differ in a single DNA-marker, possibly indicating epigenetic variation.

## Background

Vegetable oils are currently used as food, feedstock for the chemical industry and as liquid biofuels (including biodiesel). The demand for vegetable oils for bio-fuel production has increased enormously in recent years due to increased costs and instable and finite supplies of fossil fuels, and the desire to reduce greenhouse gas (GHG) emissions. In addition to traditional oilseed crops, a number of new species are now being explored for the purpose of bio-fuel production. Jatropha (*Jatropha curcas* L.) is one of these new species and has received much attention as a source of renewable oil for the production of sustainable and affordable biofuels. Despite the recent interest in jatropha, it essentially still is a wild species that has not benefitted yet from programmes of crop improvement. The agronomy of the species, now treated as an agricultural crop, is still poorly understood. This sudden boom in jatropha has therefore led to an unbalanced development, with a fast implementation of large plantations and processing units, while essential questions around jatropha crop growth, crop management and production have not been addressed adequately. Wild jatropha accessions were used to setup plantations, often not well adapted to local environments and local production systems. Maladaptation of jatropha accessions to the new use has often led to inadequate seed and oil yields per hectare. The challenge is to develop well adapted, robust, high yielding jatropha varieties for a range of climates and agrosystems, since only high seed and oil per hectare will guarantee a good profitability and a high GHG emission reduction [1]. Wide genetic variation is required in breeding for major agronomically important traits like seed and oil yield, seed and oil composition, flowering behaviour, tree morphology, disease resistance and the absence of anti-nutritional factors that currently block the use of jatropha seed meal in animal feeding. Plant breeding programs need such genetic variation to be able to combine positive traits from different parents to provide the required profitable and sustainable jatropha varieties of the future.

Jatropha is a perennial tree or shrub that produces fruits containing seeds rich in oil [2]. It grows in semi-arid tropical and subtropical climates, does not tolerate frost, and flowers only under specific temperature, radiation and phenological conditions [3]. The oil and derivatives of the oil are very suitable as a bio-fuel [2,4]. Most simply, the oil can be used without modification in the form of pure plant or vegetable oil to fuel stationary diesel engines. If the oil is esterified with methanol, the resulting methyl esters of jatropha oil form bio-diesel, which can replace or be mixed with fossil oil based diesel.

Not much is known about genetic diversity in *Jatropha curcas* and this hampers breeding of jatropha towards varieties with higher value as energy crop and with better adaptation to different forms of abiotic and biotic stresses. Before its use as a bio-energy crop, jatropha was used for medicinal products, and as a live fence around arable land. Because the plant is toxic, animals do not eat the plant. Therefore, a dense jatropha hedge keeps animals out of arable land and protects arable crops against animal grazing. The plant was also used to obtain plant oil for the production of soap [5]. For these traditional purposes naturally occurring ecotypes were used. Only recently, the use of *jatropha* as a bio-energy crop has started on the basis of such existing ecotypes without any plant breeding for bio-energy production related traits. With respect to bio-energy production, jatropha still has to be considered an undomesticated wild species [6].

Genetic diversity in *Jatropha curcas* was found to be very low in Asian, African and South American (Brazilian) germplasm [7-10]. Tang *et al.*[9] used a set of six amplified fragment length polymorphism (AFLP) primer combinations that yielded 362 AFLP-markers to analyse genetic variation in Asian *J. curcas* accessions and found low genetic variation in material from China. Also in South America, the reported genetic variation is limited [10]. South and Central America have been reported as centres of biodiversity and possible centres of origin for *J. curcas*, since it is believed that jatropha was native in America only. The Portuguese collected jatropha in America and took the plant to Cape Verde, South-Africa, Madagascar, India and finally to Indonesia. It is conceivable that only a very low number of genotypes of jatropha was collected and transferred to Africa and Asia and that this is the cause of the low level of genetic variation in Africa and Asia. If this is true, it is expected that genetic variation in South and Central America is much higher than in Asia and Africa. However, only few studies have reported the extent of genetic variation of jatropha germplasm from all these continents simultaneously [11,12]. Recent studies on genetic diversity have found high genetic variation in material from Chiapas Mexico, which shares a border with Guatemala, indicating high genetic variation in this region [11,13]. Genetic diversity in this species has mainly been analysed at the molecular marker level. It is much more interesting to relate relevant traits for bio-energy production to the molecular variation, but detailed analyses on this are lacking so far.

In this study, we analysed the genetic variation in the collection of the Jatropha curcas Evaluation Programme (JEP, [14]) in order to identify new genetic variation to be used in breeding programs of jatropha. The JEP collection contains 182 accessions from Asia (91), Africa (35), South-America (9) and Central America (47). The analysis of genetic variation included analysis of molecular marker variation, variation in seed traits (oil content and fatty acid composition), and early growth traits.

## Results

### Molecular variation

Using a set of SSRs [15,16] in the JEP collection, polymorphisms for 14 SSRs were found. Using TRAP-PCR with 13 (single) SSR-primers from non-polymorphic SSRs, 6 additional polymorphisms were identified. AFLP analysis of the JEP collection yielded 86 polymorphic bands with 2 primer combinations. The polymorphic SSRs, TRAP-primers and AFLP yielded 190 polymorphic DNA-markers among the accessions in the JEP collection (Table 1).

**Table 1:** Summary statistics for SSR, TRAP and AFLP markers

**Characteristic**	**SSR**	**TRAP**	**AFLP**
Number of markers tested for amplification	29	13	20
Number of markers yielding polymorphic patterns	14	6	2
Total number of polymorphisms amplified	73	31	86
Average number of polymorphic bands per marker	5	5	
Highest number of polymorphic bands per marker	12	10	
Lowest number of polymorphic bands per marker	2	2	
Total number of null alleles	2		
Total number of exclusive alleles	22		

### Allele frequencies and PIC values in SSR makers

Using the published SSR-primers we found the same fragment lengths as reported in literature. The percentage of SSRs with polymorphisms was 32% in the set of accessions from Africa, 58% for the set from Asia, 79% for the set from South America and 89% for the set of accessions from Central America. The mean number of alleles per polymorphic SSR was for 4.1 for Africa, 2.2 for Asia, 2.0 for South America and 3.8 for Central America. The PIC (Polymorphism Information Content) values from the different SSR markers were higher in the set of Central American accessions (Table 2).

**Table 2:** PIC values for the SSR markers between the geographical regions of Central and South America, Asia and Africa

**No**	**SSR**	**Africa**	**Asia**	**Central America**	**South America**
1	Jc01 A	0.09	0.02	0.39	0.15
2	Jc01 B	0.10	0.00	0.19	0.15
3	Jc01 C	0.12	0.02	0.33	0.15
4	Jc03 A	0.00	0.00	0.46	0.00
5	Jc05 A	0.00	0.00	0.42	0.00
6	Jc07 B	0.00	0.08	0.65	0.00
7	Jc08 A	0.00	0.00	0.26	0.00
8	Jc09 A	0.05	0.00	0.63	0.15
9	Jc10 A	0.00	0.00	0.06	0.00
10	Jc10 B	0.00	0.00	0.35	0.00
11	Jc13 A	0.09	0.00	0.56	0.15
12	Jc14 B	0.00	0.08	0.65	0.00
13	Jc17 A	0.37	0.38	0.37	0.37
14	Jc28 A	0.00	0.00	0.54	0.00

### Genetic structure of JEP collection related to region of origin

**Figure 1:**
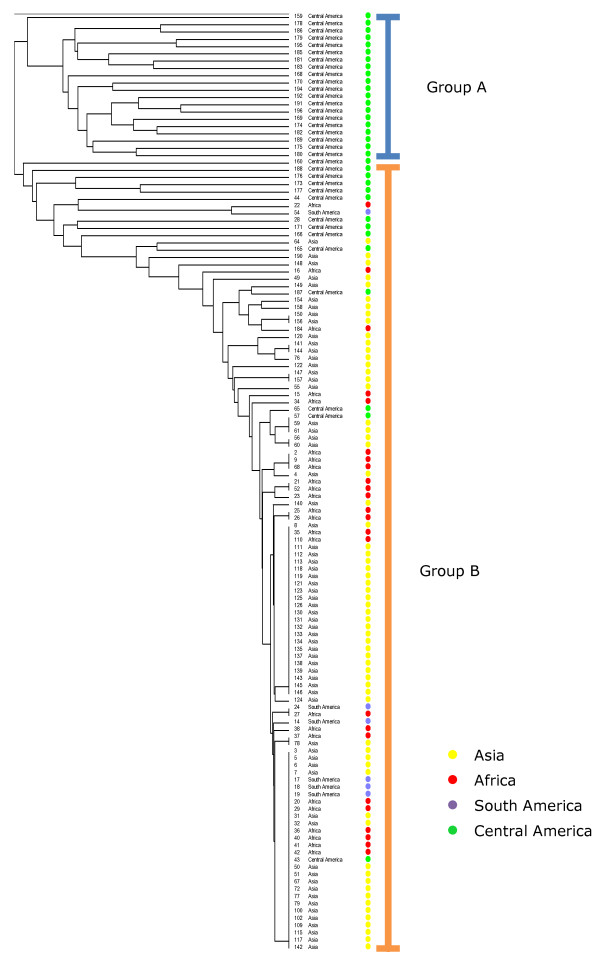
**UPGMA cluster analysis of 133 J. curcas accessions of the JEP germplasm collection using the Jaccard’s similarity index.** Colours indicate the origin of the accessions. **Groups A and B** were indicated by structure 2.3 (k = 2).

The markers scores of 190 DNA markers were used to determine the genetic distances between 182 accessions in the JEP collection using Jaccard’s coefficient and UPGMA clustering analysis. The average Jaccard’s similarity coefficient was 0.15 (of all pairwise combinations), indicating high genetic diversity in the JEP collection. Using the genetic distance, a neighbour joining tree was constructed that groups genetically similar accessions and separates genetically dissimilar accessions (Figure 1). A group of 70 accessions, mainly from Asia and Africa, did not show molecular polymorphisms for any of the 190 DNA markers for which the other accessions were polymorphic, which indicates that these accessions are genetically identical for these DNA markers. The other accessions from Asia, Africa and South America showed more polymorphisms, but are nonetheless highly genetically similar to the group of 70 accessions that were genetically identical. In contrast, a high level of polymorphism with these DNA markers was found for the accessions from Central America (Figure 1).

Fst-values indicated that the groups of South American and Asian accessions hardly differ genetically, but a moderate level of genetic difference was found between the groups of Asian and African accessions (Table 3). This is not surprising in view of the large number of Asian, African and South American accessions without any polymorphisms for the DNA markers analysed. Fst-values between Central American accessions and other regions (Asia, Africa and South America) showed large to moderate genetic differentiation. AMOVA results were significant (P < 0.0001) and indicated a high percentage of genetic variation within geographical regions (81.7%) and a much lower extent of genetic variation across regions (18.3%).

**Table 3:** Genetic distance between groups

**Regions A**	**Region B**	**Fst-value**	**Significant**
Asia	Central America	0.312	***
South America	Central America	0.119	**
Africa	Central America	0.103	**
Asia	Africa	0.092	*
Africa	South America	0.046	*(ngd)*
Asia	South America	0.023	*(ngd)*

^***^Fst > 0.15 indicates large genetic differentiation.

**Fst between 0.10 and 0.15 indicates moderate genetic differentiation.

*Fst between 0.05 and 0.10 little genetic differentiation.

(ngd) Fst < 0.05 indicates negligible genetic differentiation.

Fixation index (Fst) between the geographical regions of Central and South America, Asia and Africa.

**Figure 2:**
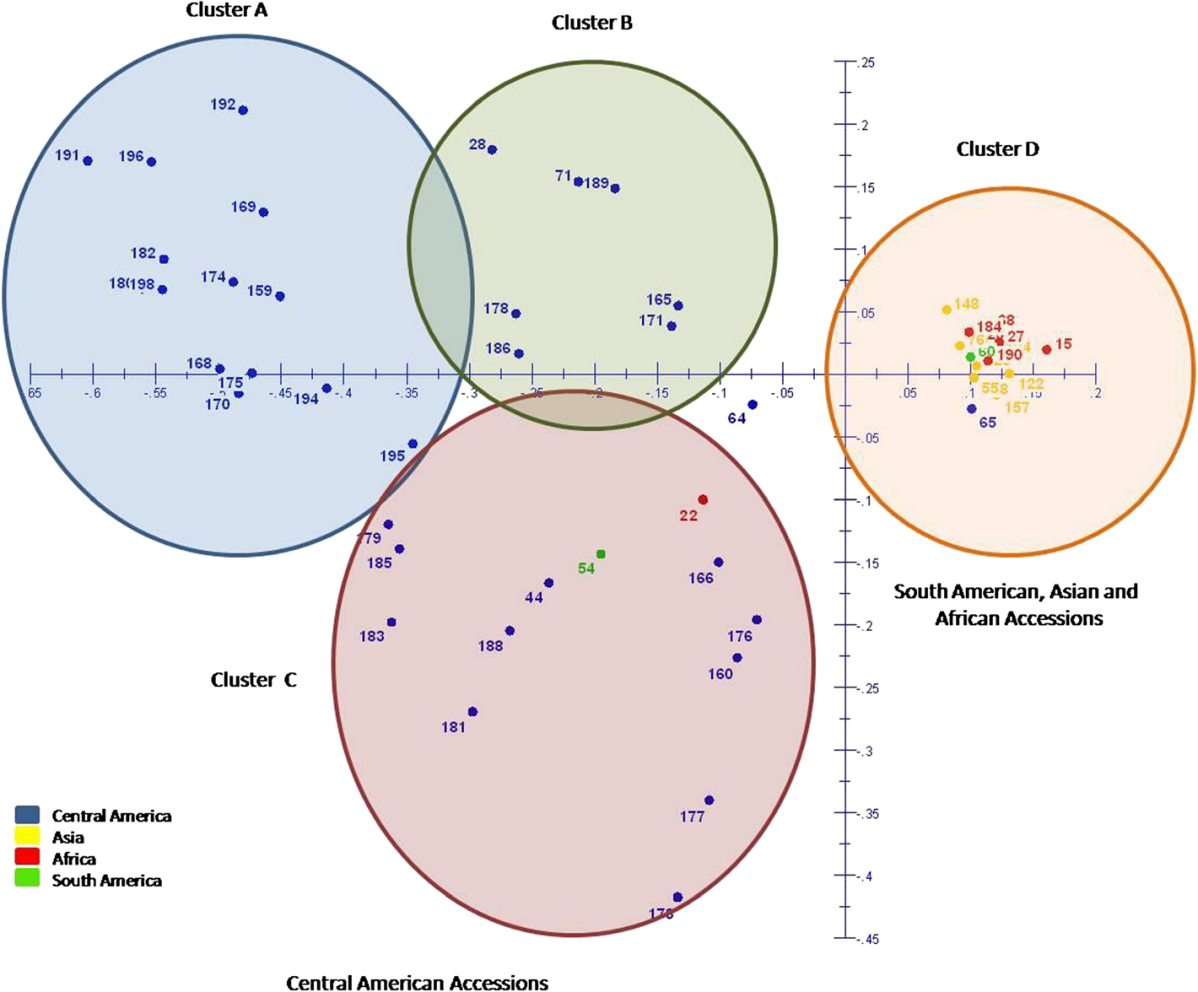
**PCA scatter plot for J. curcas accessions of the JEP germplasm collection. Cluster A** and **B** (Central Amercia accessions), **Cluster C** (Central America, Africa and South America accessions), **Cluster D** (Central America, Asia, Africa and South America accessions).

PCA on the basis of the DNA-marker data shows a clear separation between accessions from Central America and the ones from Africa, Asia and South America (Figure 2). The PCA shows four different clusters. The accessions from Central America are separated into three highly differentiated clusters (A, B and C). Most of the accessions from Africa, Asia and South America occur in one single cluster (D). Cluster A mainly contains accessions from the South and South East regions of Guatemala. Cluster B has a mixture of accessions from the northern and southern regions of Guatemala. Cluster C has mixture of accessions from Central America with one South American and one African accession, and cluster D contains the majority of accessions from Africa, Asia, South America, and only very few from Central America.

The analysis of the population by structure 2.3.2 [17] indicated two main populations (k = 2) can be distinguished, which are visualized in the cluster analysis in Figure 1. One group exclusively contains accessions from Central America and the other group contains accessions from Central America, Asia, Africa and South America.

### Seed and seedling traits

#### Seed weight, seed hull and seed oil content

The average seed weight of the accessions in the JEP collection ranged from 0.4 to 0.9 g per seed. Seed hull percentage ranged between 32%. and 52%. The average oil content in the seed (w/w) of the accessions varied between 19 and 40% of the whole seed (seed kernel and seed hull). The average seed oil content of all accessions was 28% with no significant differences between the regions.

#### Seed oil fatty acid composition

Fatty acid composition of the seed oil showed large variation in the JEP collection. The content of palmitic acid (C16:0) showed significant differences between regions (P < 0.001); accessions from South America showed the highest percentages (15.4%), followed by accessions from Africa (15.0%), Asia (14.8%) and Central America (13.6%) (Table 4). The content of stearic acid (C18:0) did not show significant differences between regions (P > 0.05). Palmitoleic acid (C16:1) contents were very low, but the small differences between regions were statistically significant (P < 0.05). Accessions from Asia, Africa and South America showed similar values between 42.0-46.1% of oleic acid content (C18:1), whereas accessions from Central America showed significantly lower C18:1 content (only 34.5%). The linoleic acid content (C18:2) of accessions from Central America was significantly higher (43.1% on average) than that of accessions from Asia, Africa and South America (30.5%, 34.6% and 33.1% respectively). α-Linolenic acid (C18:3) levels were very low (0.2%) for all regions (Table 4). The ranges of fatty acid contents of C18:1 and C18:2 were high in all regions (for the whole collection ranging from 24.1 to 53.8% for C18:1 and 22.0 to 52% for C18:2, Table 4C), but these ranges were highest for Central America. This is also reflected in the higher coefficients of genetic variation (CVg%) in Central America than in the other regions for C18:1 and C18:2. The sum of C18:1 and C18:2 was rather constant at about 78%.

**Table 4:** Fatty acid composition between jatropha accessions from different regions

**A. Fatty acid composition of seed oil (% of total fatty acids)**				
**Fatty acids**	**Asia**	**Africa**	**South America**	**Central America**
C16:0*	14.8a	15.0a	15.4a	13.6b
C16:1*	0.7a	0.7a	0.7a	0.6b
C18:0(ns)	8.2	8.0	8.6	8.4
C18:1*	46.1a	42.0a	42.9a	34.5b
C18:2*	30.5a	34.6a	33.1a	43.1b
C18:3(ns)	0.2	0.2	0.2	0.2
* = variation between accessions is statistically significant (p < 0.05). (ns) = no significant differences (p > 0.05). Differences between regions significant when denoted with different letters.				
**B. Coefficient of genetic variation (CV**_**g**_**, standard deviation of set of accessions divided by the mean, as%) of fatty acid contents**				
**Fatty acids**	**Asia**	**Africa**	**South America**	**Central America**
C16:0*	4.4	6.7	10.4	4.3
C16:1*	11.1	8.2	14.5	5.6
C18:0(ns)	6.3	18.3	12.6	4.6
C18:1*	9.4	9.4	8.4	17.4
C18:2*	14.0	13.0	9.9	12.9
C18:3(ns)	6.4	4.0	8.4	0
**C. Range of fatty acid contents (% of total fatty acids)**				
**Fatty acids**	**Asia**	**Africa**	**South America**	**Central America**
C16:0*	12.4-17.5	13.1-16.9	10.5-17.1	11.3-16.6
C16:1*	0.5-1.1	0.6-0.9	0.4-1	0.4-0.9
C18:0(ns)	5.5-11.3	6.1-13.4	5.7-10.3	6.1-10.4
C18:1*	31.0-53.8	34.2-52.1	35.9-49.5	24.1-50.7
C18:2*	22.0-43.3	24-43.3	29.3-40.1	25.2-52
C18:3(ns)	0.1-0.2	0.1-0.3	0.1-0.2	0.1-0.2
* = variation between accessions is statistically significant (p < 0.05). (ns) = no significant differences (p > 0.05). Differences between means of regions are significant when denoted with different letters (Table 4A).				

A. Fatty acid percentage. B. Coefficient of variation of the fatty acid between regions. C. Fatty acids range (Maximum and Minimum values) in different regions.

#### Seedling growth and morphology

Significant and large genetic variation was found between accessions in the JEP collection for almost all of the observed early growth and morphology traits (Table 5). A fast early growth is very beneficial as it is one of the factors positively influencing the yield of seed and oil in the first year of establishment. A positive correlation (r > 0.83) was found between all biomass variables (root, stem, leaf, petiole and total plant dry weight) and plant height, first leaf length and width and total leaf area and absolute growth rate. The broad sense heritability (h^2^) of most traits was high (50–90%), except for cotyledon number and petiole weight (Table 5). Table 6 shows the variability for phenotypic traits between the regions in the JEP collection. Central American accessions, on average, had the highest total growth rates (indicated by the higher dry weights 59 DAG). Also, for most traits, the coefficient of genetic variation was highest in the set of Central American accessions. Especially for total leaf area and for root and petiole dry weight, but not for total above ground dry weight for which the coefficient of genetic variation was not highest in Central America.

**Table 5:** Phenotypic variation in J. curcas among accessions

**Trait**	**Mean**	**Min.**	**Max.**	**SD**_**g**_	**CV**_**g**_**%**	**h**^**2 **^**(%)**
Average seed weight (g seed^−1^)	0.66	0.44	0.89	0.089	13	n.d.
Cotyledon number (#)	2.02	2	3	0.009	0.4	1.4
Days to cotyledon emergence (d)	18.2	14.5	28.0	2.1	12	88.3
Days to germination (d)	14.8	11.0	22.0	1.7	12	74.8
Plant height (cm)	21.3	7.5	33.4	3.5	17	85.7
Leaf number (#)	12.0	7.0	17	0.8	7	48.0
Phyllochron in days per leaf (d)	5.1	2.7	8.3	0.44	9	64.3
First-leaf length (cm)	12.6	7.2	20.3	1.6	13	81.9
First-leaf width (cm)	11.9	7.2	19.8	1.9	16	83.4
Leaf area average (cm^2^ leaf^−1^)	173	112	327	35	20	87.8
Root dry weight (g plant^−1^)	1.3	0.4	2.5	0.29	23	70.0
Petiole dry weight (g plant^−1^ ) ns	1.9	0.8	3.0	0.20	10	39.2
Stem dry weight (g plant^−1^)	6.6	2.2	12.5	1.39	21	71.2
Leaf dry weight (g plant^−1^)	6.4	3.2	10.4	0.87	14	59.2
Total plant dry weight (g plant^−1^)	16.1	7.5	27.6	2.7	17	66.4
Total leaf area (cm^2^ plant^−1^)	2044	1044	3158	303	15	75.5
Absolute growth rate (g d^−1^)	0.27	0.13	0.47	0.046	17	67.5
Relative Growth Rate, RGR (d^−1^)	0.053	0.040	0.060	0.002	4	47.4
Leaf Weight Ratio, LWR (%)	40.1	34	55	2.0	5	62.8
Specific Leaf Area, SLA (cm^2^ g^−1^)	324	220	416	22	7	64.7
Net Assimilation Rate, NAR (g m^2^ d^−1^)	4.2	2.4	5.9	0.4	9	48.4
Radiation use efficiency (g MJ_int_^−1^)	5.0	2.4	8.0	0.67	13	57.3
Shoot/root ratio (−)	12.5	8.0	24.4	1.8	14	73.7
Leaves/stem ratio (−)	1.3	1.0	2.4	0.14	11	69.6
Petiole/leaf weight ratio (−)	0.30	0.16	0.38	0.031	10	78.8

No significant difference between accessions for petiole dry weights (ns).

RGR, RUE and NAR differences statistically significant at p < 0.05.

Difference for all other traits statistically significant at p < 0.01.

Note: RGR = LWR*SLA*NAR (when SLA in m^2^ g^−1^ and NAR in g m^2^ d^−1^ and LWR expressed as a fraction).

Means over all accessions, minimum, maximum values of accession means, genetic standard deviation (SD_g_) and genetic coefficient of variation (CV_g_% = 100*SD_g_/mean), broad sense heritability (h^2^, for family means based on three plants per family).

**Table 6:** Phenotypic and genotypic variability among accessions across geographical regions

	**Asia**		**Africa**		**South America**		**Central America**	
**Trait**	**Mean**	**CV**_**g**_	**Mean**	**CV**_**g**_	**Mean**	**CV**_**g**_	**Mean**	**CV**_**g**_
Germination time (d)	14.9	10.7	14.0	12.9	14.2	9.5	13.8	12.0
Cotyledon emergence date (d)	18.4	10.9	17.5	11.4	17.9	7.4	17.1	10.7
First-leaf length (cm)	12.0	7.8	12.2	5.8	13.1	7.1	14.6	14.3
First-leaf width (cm)	11.0	7.6	11.8	6.0	12.1	7.4	14.6	15.0
Plant height (cm)	20.1	12.1	20	15.6	22.1	8.5	24.9	14.1
Leaf number (# plant^−1^)	12.1	7.7	11.9	7.4	12.8	10.0	11.8	6.2
Root dry weight (g plant^−1^)	1.2	18.4	1.1	19.9	1.4	24.8	1.6	16.0
Petiole dry weight (g plant^−1^)	1.9	9.7	1.9	8.3	2.2	14.3	2.0	16.8
Stem dry weight (g plant^−1^)	6.1	14.6	6.0	19.6	7.3	16.4	8.0	14.5
Leaf dry weight (g plant^−1^)	6.0	4.1	5.8	10.6	6.8	11.0	7.5	9.3
Total dry weight (g plant^−1^)	15.1	11.0	14.5	14.6	17.4	14.8	19.1	11.7
Total leaf area (cm2 plant^−1^)	1874	6.9	1933	5.6	2139	0.0	2525	10.9

CV_g_ is the coefficient of genetic variation (SD_g_/mean). Plant height and plant weights were determined 59 days after germination.

#### Relative growth rate (RGR) and its components

**Figure 3:**
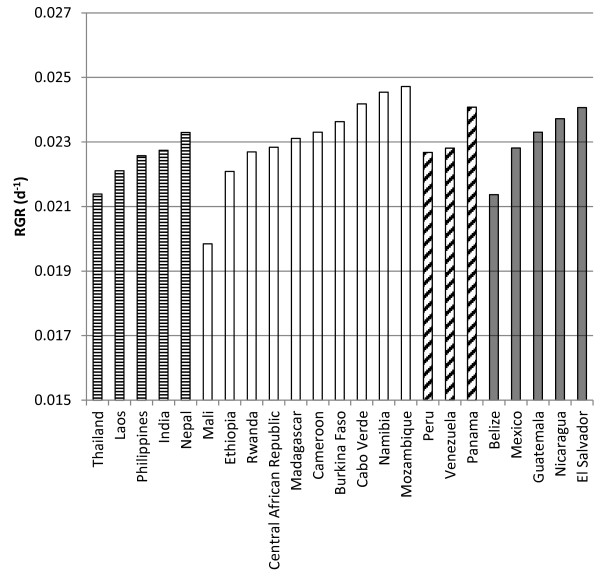
**Variation in relative growth rate (RGR, d-1) between J. curcas accessions from different countries.** Horizontal dash: Asian origin; no fill: African origin; tilted dash: South American origin; grey: Central American origin.

RGR ranged from 0.040-0.060 d^−1^ between accessions (F-test significant at p < 0.01). RGR averages per country ranged from 0.045-0.057 d^−1^ (Figure 3). No significant differences in the average RGR between the 4 regions (Asia, Africa, South America and Central America) were observed (P > 0.05) (Table 7). Significant differences were found for specific leaf area (SLA) and ranged from 220 to 416 cm^2^ g^−1^ (P < 0.001). Leaf weight ratio (LWR) also showed significant differences (P < 0.001) and ranged from 34% to 55% among all individual accessions (Table 7). Variation between accessions for net assimilation rate (NAR; g m^−2^ d^−1^) correlated highly with variation in RGR (r = 0.83) and in RUE (r = 0.95).

**Table 7:** Genetic variation between accession of J. curcas from different countries and regions

**Country**	**Region**	**Seed weight (g)**	**RGR (d**^**−1**^**)**	**SLA (cm**^**2**^ **g**^**−1**^**)**	**LWR (%)**	**NAR (g m**^**−2 **^**d**^**−1**^**)**
Thailand	Asia	0.57	0.049	275	46.0	3.9
Laos	Asia	0.59	0.051	344	44.3	3.5
Philippines	Asia	0.78	0.052	281	39.3	4.9
India	Asia	0.63	0.052	321	40.1	4.2
Nepal	Asia	0.64	0.054	315	36.1	4.8
Mali	Africa	0.59	0.046a	345	45.4	3.0
Ethiopia	Africa	0.63	0.051	354	40.6	3.7
Rwanda	Africa	0.48	0.052	366	44.7	3.2
Central African Republic	Africa	0.64	0.053	350	39.6	3.8
Madagascar	Africa	0.76	0.053	291	36.8	5.0
Cameroon	Africa	0.60	0.054	349	38.6	4.2
Burkina Faso	Africa	0.57	0.054	289	43.6	4.4
Cape Verde	Africa	0.71	0.056	297	42.1	4.5
Namibia	Africa	0.53	0.057b	320	39.1	4.6
Mozambique	Africa	0.64	0.057b	285	37.1	5.4
Peru	S-America	0.74	0.052	313	38.9	4.4
Venezuela	S-America	0.70	0.053	339	40.9	3.8
El Salvador	C-America	0.74	0.045a	381	43.5	3.3
Belize	C-America	0.74	0.049	331	41.3	3.6
Mexico	C-America	0.75	0.053	331	42.9	3.7
Guatemala	C-America	0.76	0.054	343	39.9	4.4
Nicaragua	C-America	0.79	0.055	340	39.3	4.1
Panama	C-America	0.57	0.055	326	44.5	3.9
LSD Countries (p = 0.05)		0.10*	0.007^ns^	60*	5.9*	1.3*
LSD Regions (p = 0.05)		0.02*	0.001^ns^	16*	1.6^ns^	0.4^ns^

* = statistically significant at p < 0.05.

ns = non-significant, p > 0.05; RGR differences between the top two countries (b) and bottom two countries (a) are significant in pairwise comparisons, although the overall analysis of variance shows no significant differences between countries.

RGR is the relative growth rate. SLA is the specific leaf area. LWR is the leaf weight ratio as percentage of total plant weight and NAR is the calculated net assimilation rate. For each region, the countries are sorted according to increasing RGR.

#### Relating phenotypic variation in early growth traits to molecular variation

**Figure 4:**
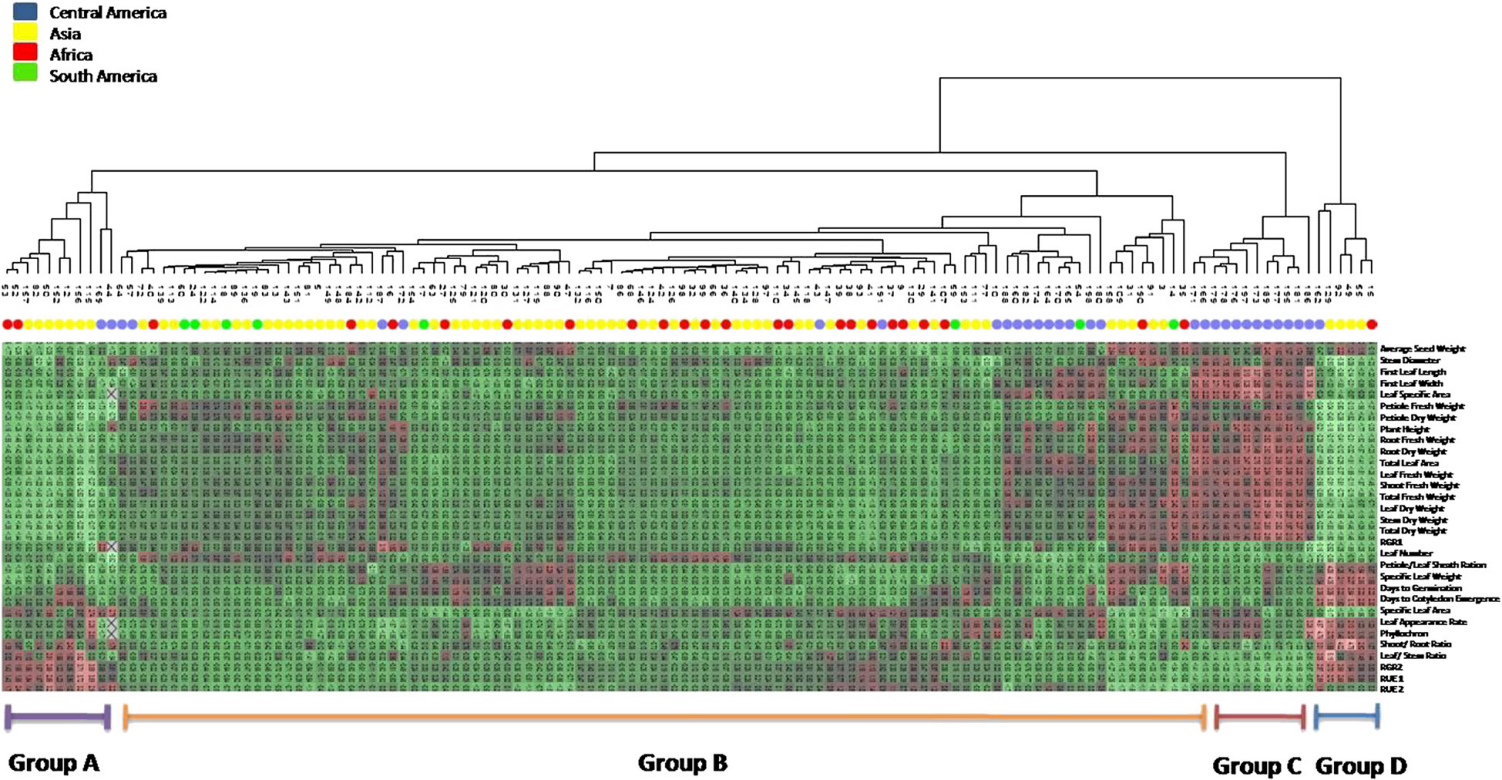
**UPGMA dendrogram of morphology traits of J. curcas accessions of the JEP germplasm collection. Group A** (Asia and Africa accession), **Group B** (Asia, Africa and South America accession), **Group C** (Central America accessions) and **Group D** (Asia and Africa accessions).

Population analysis based on phenotypic variation in early growth traits showed significant variation between accessions from the different regions. A dendrogram based on Euclidean distances showed four different groups (Figure 4). The largest group B contains the majority of accessions from Asia and Africa, all accessions from South America and few accessions from Central America. Group A and D are composed by few accessions from Asia, Africa and Central America and group C only contains accessions from Central America.

A Mantel-test between the molecular marker and the phenotypic early growth trait similarity matrices showed a low but significant (P < 0.05) correlation between the genetic and phenotypic similarity matrices indicating that the genetic structure of the JEP collection (based on molecular markers) is reflected also in the phenotypic variation (r = 0.27).

## Discussion

**Figure 5:**
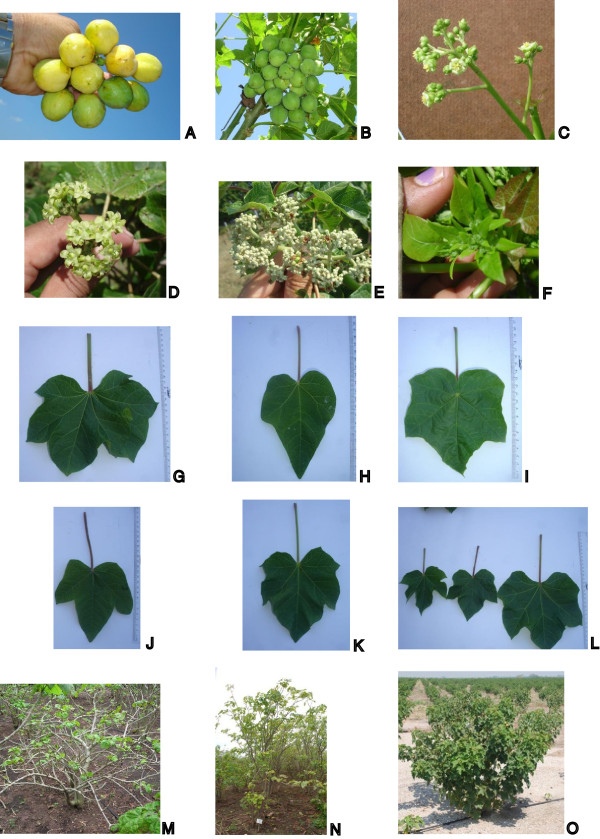
**Phenotypic variation in the JEP collection.** Variation in the number of fruit **(A and B)**. Male flower plant **(C)**. Female flower plant -type 1- **(D)**. Female flower plant -type 2- **(E)**. Bracteole inflorescence **(F)**. Different leave shapes **(G, H, I, J and K)**. Leaves size variation **(L)**. Different canopy types observed in jatropha **(M, N and O)**.

This is the first published comprehensive study of *Jatropha curcas* biodiversity among a world wide collection of accessions that assesses both molecular genetic variation nd variation in phenotypic traits. Large phenotypic variation between jatropha accessions in the world-wide JEP collection was observed in plant characteristics like early growth traits, flowering type, tree architecture and leaf shape and size. Most phenotypic variation was found among accessions from Central America (Figure 5). It was at first unknown whether this variation was only due to environmental variation or due to genetic factors.

The DNA marker analysis showed that the large phenotypic variation in the JEP collection is accompanied by a large genetic variation at the genome level. In previous studies in which some of the SSRs used here were developed, no SSR polymorphisms could be found in the (Asian) jatropha accessions [15,16]. In our study we find a high degrees of polymorphism for the same SSRs in the total JEP collection. PIC values (indicating the level of allelic variation per SSR) were higher in the set of accessions from Central America than in sets from other regions. The low PIC values for Asia found here, where the PIC-values were even 0 for some markers, confirm the low level of genetic variation in accessions from Asia previously found by others (Table 2). This was consistent with the fact that 70 accessions from Asia and Africa did not show any polymorphism for the markers evaluated.

Cluster analysis by UPGMA (Figure 1) and PCA (Figure 2) demonstrated that accessions from Asia, Africa and South America were genetically highly similar, and cluster together in both analyses. Still, AMOVA and Fst-values indicated that variation is present within accessions from all four regions, but is highest in the set of Central American accessions. Central American accessions did not show a clear geographical distribution in the different cluster analyses (Figure 1), which indicates that the Central American accessions do not form isolated populations, but can be regarded as a large inter-mating population in which a high level of genetic variation has been maintained. PCA analysis, however, showed four groups: one cluster of mainly Asian, Africa and South-American accessions and three clusters of Guatemalan accessions. These three Guatemalan clusters show three distinct genetic groups; one with partial geographical separation, but two groups contain accessions from geographical regions in Guatemala that are widely apart. This shows that different genetically distinct types of jatropha occur, but that the genetic distinction does not follow a strict geographical separation in Guatemala. The absence of a clear geographical separation between distinct types might be due to migration of farmers within Guatemala. Farmers, using jatropha as a hedge for cattle, took along jatropha cuttings and seeds when migrating to new areas [18].

Not only do accessions differ in their genetic constitution, but also show wide variation in phenotypic traits like seed hull, oil concentration and fatty acid composition. Interestingly, in the group of accessions from Asia and Africa that did not show differences at the genetic level - as inferred from the total absence of polymorphisms in DNA-markers - still variation in early growth traits and morphological traits was found. Apparent genetic differences between accessions that do not differ in DNA-marker profile have been reported in other studies from Asia for in traits like seed weight and seed oil content [19-21], The seeds of the different accessions were produced in the country of origin of the accessions and therefore environmental differences within and between the countries of origin may also have caused the differences [22]. Fatty acid composition also varied between accessions, especially with respect to the ratio of C18:1 to C18:2. The content of saturated fatty acid (SFA) and unsaturated fatty acid (UFA) in the seed oil did not differ much between the regions (ranging from 22.0% to 24.0% for SFA and from 77.5% to 78.4% for UFA). This high content of UFA was also observed in other studies, for example in accessions from Mexico with UFA percentages between 74–83% [23]. The relatively low SFA is an advantage of jatropha oil compared to palm oil as it gives a lower cloud point when making biodiesel from the oil. A too high UFA content can increase the oxidative instability of biodiesel and for that reason it is important to breed varieties with a higher C18:1 content as this has the advantage of giving a lower cloud point – enabling use of the biodiesel in colder areas of the world – and a higher oxidative stability compared to oil with highly unsaturated fatty acids [24,25]. The concentration of SFA and UFA (and the ratio of C18:1 to C18:2) are not only controlled by genetic factors, but also by environmental conditions such (e.g. temperature) and post-harvest process conditions affect the fatty acid composition. In jatropha, altitude can affect fatty composition through effects of temperature [23]. In soybean, genetic differences in the effect of temperature on fatty acid profiles have been reported [26], indicating that it may be important in jatropha to test genotypes in environments with different temperatures in order to select genotypes with a stable, desired fatty acid composition across environments.

Early growth evaluation under greenhouse condition showed phenotypic variation and high heritability values for almost all the seedling traits (50–90%). This indicates a high level of genetic variation in the variation of these traits. Surprisingly, the large group of Asian and African accessions with no or only few polymorphisms in DNA-markers, also showed a considerable variation in early growth traits. This phenomenon of highly variable growth traits is also observed in many jatropha field experiments and commercial plantations, even when seed from a single genetic source was used. A possible explanation for such phenotypic variation among accessions that do not show differences in DNA-markers might lie in epigenetic variation, for example through differences in DNA-methylation that do not lead to differences in the nucleotide sequence of the DNA, but can lead to differences in expression of the methylated genes. Such epigenetic variation has been reported in jatropha [27,28].

Although variation for early growth traits in the genetically uniform Asian accessions was found, the variation for the early growth traits was much higher in the group of Central American accessions (Table 6). The group of Central American accessions not only had the highest level of genetic variation for the traits compared to other groups, but also showed significantly higher early growth rates, resulting in higher total leaf areas, dry weights, and plant heights. These traits that lead to larger and stronger plants are important for surviving the first stages in the field after transplanting, especially under dry conditions (low precipitation <1,000 mm/year), and for taking advantage of short precipitation periods.

Specific Leaf Area values (SLA) ranged from 220 to 416 cm^2^ g^−1^ and Leaf Weight Ratio (LWR) from 34 to 55% among all individual accessions. These ranges are smaller if average values per country are calculated (Table 7). The range among accessions of the calculated Net Assimilation Rate (NAR) was large: 2.4-5.9 g m^2^ d^−1^(Table 5). NAR was highly positively correlated with the Relative Growth Rate (RGR). SLA and LWR were negatively correlated to RGR and NAR. Differences in SLA explained 19% and differences in LWR explained 40% of the variation in RGR. A highly negative correlation between SLA and NAR was found (r = −0.82). A negative correlation between NAR and SLA has also been reported in other plant species [29,30]. A high SLA implies a thinner leaf and a lower density of nitrogen and chlorophyll per area of leaf. Therefore, the photosynthetic capacity per leaf area for plants with low SLA is usually lower than for plants with a higher SLA. The calculated NAR reflects such differences in photosynthetic capacity to assimilate CO_2_. A high NAR is beneficial, as a higher photosynthesis rate is the basis for a higher growth rate at the same light interception fraction. A high SLA can also be beneficial in situations with lower light levels, for instance in intercropping systems where different species are combined and competition for light may be present. A plant with a high SLA (thin leaves) may expand its leaf area at a higher rate than a plant with a lower SLA, as it needs less dry matter to produce the same amount of leaf area, leading to a higher light interception capacity. This study shows that in *J. curcas* genetic variation occurs in both SLA and NAR, but also shows the trade-off between the two, as expressed in RGR (See Eq. 2).

**Figure 6:**
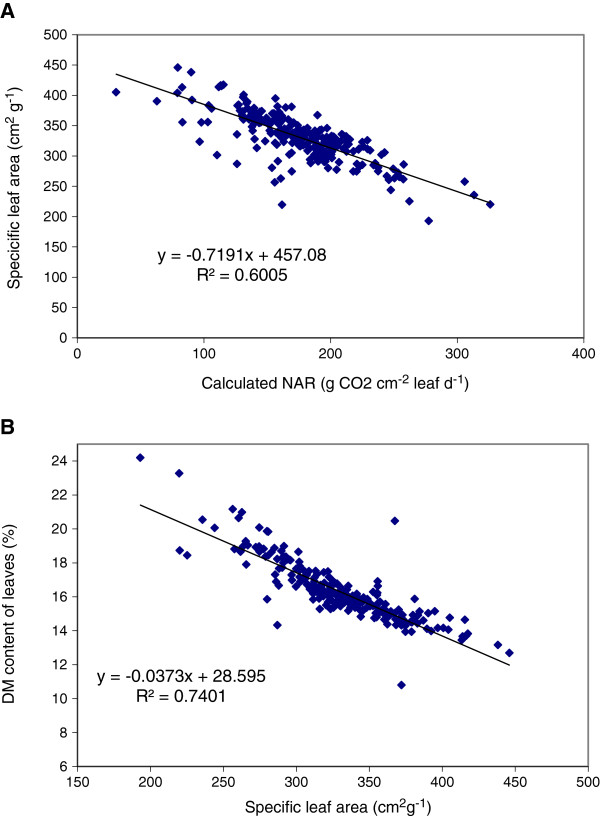
**JEP germplasm collection of jatropha. (A)** Relation between the specific leaf area and the calculated net assimilation rate (NAR) and **(B)** Relation between the dry matter content of leaves and the specific leaf area.

SLA proved to be highly negatively correlated with dry matter content of leaves (Figure 6). This relation is not fully unexpected. The amount of leaf area per amount of leaf dry matter reflects two aspects of leaf morphology: the thickness of the leaf and the density of the leaf. The density of the leaf is the amount of dry matter per volume of leaf. At the same density, leaf thickness and SLA will show a strictly positive and linear relation. At the same thickness, density and SLA will be positively related. One cause of variation in density is variation in the water content of the leaf. Clearly the density of dry matter increases when the dry matter content of the leaf increases. Apparently, such variation in density is present in the studied set of accessions under greenhouse conditions, and this variation in density caused associated changes in SLA. A high level of genetic variation was found for RGR and its components SLA, LWR and NAR in the JEP collection. In order to interpret the impact of an increase in RGR by plant breeding better, it will help to realise that an increase of only 10% in RGR can result in a difference of 20% in absolute dry matter accumulation in 60 days. Such improvements in early growth would be large enough to justify selection for early growth in breeding programs of *Jatropha curcas*.

Our results clearly indicate that outside Central America (including Mexico) there is no significant genetic variation in *Jatropha curcas*. This is supported by similar results from Chiapas Mexico (sharing a border with Guatemala), where high genetic variation was found in *Jatropha curcas*[23]. In other studies with accessions from Asia, Africa and South America, low to moderate variation was found [8,10,15,16,31]. These results also support the hypothesis that jatropha was probably distributed by Portuguese seafarers from Central America, through the Caribbean via The Cape Verde Islands to other countries in Africa and to Asia [5].

## Conclusions

In this study we found a high level of genetic variation in *Jatropha curcas* both in terms of DNA-marker polymorphisms and in phenotypic traits. The analysis of genetic variation in the JEP collection showed that variation in jatropha is concentrated in Central American accessions. The results obtained from the JEP collection also showed that accessions from Asia and Africa without genetic variation for the markers evaluated in this study still show phenotypic variation. Possibly, the phenotypic variation is associated with genomic areas not covered by the DNA-markers used in this study, but it is very likely that epigenetic variation exists in the genetically uniform Asian and African accessions that causes part of the phenotypic variation. Central American accessions always showed a much higher genetic and phenotypic variation for the seed traits and early growth traits analysed than accessions from other regions. This makes Central America an important source of new genetic variation in jatropha that will prove useful in widening the genetic variation available to jatropha breeding programmes.

## Methods

### Plant materials: the collection of the global Jatropha evaluation programme

In the Global Jatropha Evaluation Programme (JEP; [14]), a collection of 182 *Jatropha curcas* accessions was established from 26 countries. The collection contains 47 accessions from Central America, 9 from South America, 35 from Africa and 91 from Asia (Table 8). Asia is highly overrepresented in the collection, and within Asia, India is overrepresented with 73 accessions. This overrepresentation is partly accidental, as the collection of accessions was based on a general request to many organisations in different countries involved in jatropha research and growth and the contribution of accessions was voluntary. Although the reported genetic variation in Asian accessions is low, we decided to include all Indian and other Asian accessions in this study to increase the probability of finding genetic variation.

**Table 8:** Regions and countries of origin of jatropha accessions collected for the Global Jatropha Evaluation Programme (JEP)

**Country**	**Number of accessions**	**Region**
India	73	Asia
Indonesia	1	Asia
Laos	7	Asia
Nepal	4	Asia
Philippines	5	Asia
Thailand	1	Asia
Burkina Faso	3	Africa
Cabo Verde	1	Africa
Cameroon	3	Africa
Central African Republic	2	Africa
Ethiopia	9	Africa
Gambia	1	Africa
Madagascar	1	Africa
Mali	3	Africa
Mozambique	3	Africa
Namibia	5	Africa
Rwanda	4	Africa
Peru	7	South America
Venezuela	2	South America
Belize	2	Central America
El Salvador	1	Central America
Guatemala	39	Central America
Honduras	1	Central America
Mexico	1	Central America
Nicaragua	2	Central America
Panama	1	Central America

### Analysis of molecular genetic variation

#### DNA isolation

Seed samples from all 182 accessions of the JEP germplasm collection were germinated and grown in order to obtain fresh plant material for DNA isolation, under the following greenhouse conditions:Tmin. 20°C; Tmax 25°C; relative humidity between 50–80%; photosynthetically active radiation (PAR) 3 MJ m^−2^ d^−1^ ( about 35 W m^−2^). For all germinated accessions, a young leaf sample of approximately 2 cm^2^ was ground in 800 μL of preheated (65°C) extraction buffer [32]. The 182 samples of homogenized ground samples were incubated at 65°C for 1.5 h, and stirred every 15 min. Samples were then treated with 10 μL RNAse [20 mg/mL, Invitrogen (Carlsbad, California)] and incubated at 37°C for 30 min. Subsequently, 1 mL of chloroform-isoamyl solution was added to each sample and centrifuged for 5 min at 14,000 rpm in order to separate an aqueous and solid phase. The aqueous phase was then transferred into a new tube, and total genomic DNA was recovered from this supernatant by using the “Mini-Spin Columns” and DNA purification protocol of the DNeasy Plant Mini-Kit (QIAGEN, Valencia, California). DNA concentrations were measured using the Nanodrop ND-1000 Spectrophotometer (ISOGEN Life-science, Maarssen, Netherlands), and all samples were finally diluted to a concentration of 20 ng/mL of DNA.

#### Simple sequence repeat markers

Twenty-nine jatropha-specific simple sequence repeat (SSR) markers [15,16] were tested for amplification quality and polymorphic content on six different accessions of the JEP collection. Only polymorphic SSR from this evaluation were used in the complete JEP collection. SSR markers were amplified in a 10 μL polymerase chain reaction (PCR) reaction containing 30 ng of DNA template, 50 mM KCl, 20 mM Tris–HCl (pH 8.4), 1.5 mM MgCl_2_, 0.20 nM Primer Mix (Forward and Reverse), 0.20 mM dNTPs, and 1.5 U SuperTaq® Polymerase. For all SSR primer pair combinations, the reverse primer was labelled with near-infrared fluorescence (IRDye 700). The labelled products were separated by electrophoresis on 6% (g/mL) denaturing polyacrylamide gels using a Li-COR IR2 DNA Analyzer (Li-COR, Lincoln, New England). SSR markers that were not polymorphic were used in the target region amplification polymorphism marker system (TRAP). Bands scoring was performed visually by evaluating only the strongest bands as alleles, since lighter or blurry bands could be stutter bands, a by-product of DNA Taq polymerase slippage. All bands were scored as presence/absence dominant markers, and a binary raw-data matrix was generated for all gel banding patterns, in which a positive allele (presence of a band) was encoded as 1 and a negative allele as 0 (absence of a band), The molecular size of the bands was determined to enable comparison to the molecular sizes of the SSR fragments reported previously.

#### TRAP markers

The non-polymorphic SSR primers were used in the target region amplification polymorphism (TRAP) system [33]. These SSR were combined in a PCR reaction with an “arbitrary primer” which has a random sequence with either an adenine-thymine or guanine-cytosine (AT- or GC) rich core. This gives it an affinity to gene intron and exon sequences [33]. The main goal of the technique is to generate a large number of markers and polymorphisms around target loci that are monomorphic [33]. In this study, the TRAP technique was used to produce a large number of polymorphic markers around SSR loci yielding stable monomorphic patterns. Unlabelled forward primers for all selected SSR sequences were used as fixed primers. The arbitrary reverse primers were obtained from the work of Faris *et al.*[34]. Since Li-COR DNA sequencers can simultaneously detect two fluorescent signals (at 700 and 800 nm), TRAP-based PCR reactions were run using two arbitrary primers with different fluorescent labels. The PCR reactions were carried out in a 10-μl volume containing 60 ng genomic, 50 mM KCl, 20 mM Tris–HCl (pH 8.4), 2.5 mM MgCl_2_, 0.1 mg/mL BSA (New England Biolabs, Ipswich, Massachusetts), 0.50 nM Fixed Primer, 0.20 nM Arbitrary Primer 1 (IRD-700), 0.20 nM Arbitrary Primer 2 (IRD-800), 0.20 mM dNTPs, and 1.0 U Goldstar® Taq Polymerase (Eurogentec, Seraing, Belgium). The PCR was performed by an initialcycle of denaturing the genomic DNA at 95°C for 2 min, followed by a cycle of 95°C for 45 s, 35°C for 45 s, and 72°C for 1 min, 30 cycles of 95°C for 45 s, 50°C for 45 s, and 72°C for 1 min, and a final extension period at 72°C for 7 min. All amplifications were performed on a Veriti 96 Well Thermo Cycler (Applied Biosystems) and amplified products were electrophoresed on 6% polyacrylamide gels using a Li-COR IR^2^ DNA sequencer (Li-COR). Band patterns were scored as presence/absence dominant markers with aid of the Quantar Pro band analyser software (Keygene, Wageningen, Netherlands), and independent replicates were performed for several TRAP-marker combinations in order to verify and validate band-scoring results.

#### AFLP makers

AFLP was carried out as described by Vos *et al*. [35]. Genomic DNA (250 ng) was restricted with enzymes EcoR1 and Msel at 37°C for 2 hours and the digested aliquot was ligated to EcoRi and MseI specific adapters at 20°C for 2 h (AFLP® Core Reagent Kit, USA). The ligated DNA was preamplified using EcoRI and MseI with one selective nucleotide at the 3′ end primer each. The preamplified product was diluted 1:20 with sterile tris- ethylenediaminetetraacetic (TE) buffer. The diluted product was amplified with selective primers for the EcoRI and MseI adapters withthree selective nucleotides at the 3′ end. PCR was performed using 65°C for the first cycle and subsequently for 11 cycles; the annealing temperature was successively reduced by 0.7°C. This was followed by 23 cycles at an annealing temperature of 56°C. All amplifications were performed on a PTC-200 96 Well Thermo Cycler (MJ Research-Bio-Rad). Before loading the PCR products into the electrophoresis gel an equal amount of formamide dye was added. PCR products with formamide dye were subjected to electrophoretic separation on 6% denaturing polyacrylamide gel in 1X tris-boric acid- ethylenediaminetetraacetic (TBE) buffer in a sequencing gel system (Bio-Rad Sequi-Gen GT Sequencing Cell). The gels were stained with silver nitrate using a silver staining kit (Sigma, USA). Evaluation of each primer combination of *Eco*RI and *Mse*I was done two times with three samples (two from Central America and one from Asia) to select the polymorphic and reproducible primer combinations for the complete analysis. Only clear bands between 700 and 50 bp were scored with a scale to describe the intensity of the band from 1, 2 and 3 (weak, medium and strong), and only the bands with score of 3 were used for the analysis.

#### Data analysis molecular markers

Generated SSR, TRAP and AFLP fingerprints were individually scored and statistically analysed as dominant markers. (present = 1 and absent =0). Bands of similar size and intensity were assumed to be homologous. Jaccard’s similarity coefficient [36] was used to estimate the genetic similarity (GS). Cluster analysis using Unweighted Pair Group Method with Arithmetic Mean (UPGMA) clustering was carried out with the combined binary data from SSR, TRAP and AFLP in GeneMaths XT (Ver. 1.6.1) (Applied Maths BVBA 2005). An analysis of molecular variance (AMOVA) was performed - with 10,000 permutations using the software Arlequin© version 3.11 - to determinate the degree of differentiation within and between populations. The genetic distance between the groups of accessions from different regions was expressed in terms of the Fixation Index (Fst). Fst is a measure of the allelic variation within and between groups of accessions. If for two groups Fst equals 0, these groups have the same allele frequencies for all markers, and this indicates that the two groups are freely interbreeding, and effectively form one genetic group. If Fst equals 1, the two groups are genetically fully separated. Genetic structure of the populations was analysed using the software Structure© version 2.3.2. This software was run with 30,000 iterations, 50,000 iterations after burn-in and 10 repetitions of each number of genetic populations (K1-K9) [11]. To determinate correlation between phenotypic and molecular data, a Mantel test was done using the similarity matrix from phenotypic and molecular marker in GenStat 11.0^th^[37,38]. ANOVA analysis was done to determine significant differences between markers and traits in GenStat 11.0^th^[37]. PIC values, heterozygosity and allele number were also calculated from the SSRs’ markers using the microsatellite tools kit for MS Excel [39].

### Analysis of seed and seedling traits

#### Seed hull percentage

Four seeds of each accession were randomly selected from each accession for analysis of total seed weight and weights of seed hull and kernel. The seeds were dried at 70°C for 24 h to obtain a stable dry matter content of more than 96%. Each individual seed was mildly crushed to facilitate the separation of seed hull and kernel. Seed hull and kernel were weighed per individual seed.

#### Oil content

The determination of oil content was carried out on individual kernels. For this, we first crushed the kernel, which weighed between 0.3 and 1.0 g, to a fine powder. The whole powder sample was used to extract the seed oil using three consecutive cycles of hexane extraction. In the first cycle of hexane extraction, the sample was added to a 15 ml tube to which 7.5 ml of hexane was added. The tube was thoroughly shaken using a vortex during 10 s to homogenize the mixture of hexane and powder sample. The tubes with this mixture were placed on a shaking plate that stirred the tubes at 250 rpm for 30 min to allow an efficient extraction of the oil into the hexane. After shaking, the tubes were centrifuged at 4000 rpm for 10 min, and the supernatant containing hexane and extracted seed oil was carefully transferred to glass tubes of 10 ml. The hexane was evaporated from the hexane-oil mixture to obtain pure seed oil extracts using a Rapid Vap® Vacuum Evaporation System (Labconco, Kansas City, Missouri); a run time of 1 h at 30°C was used. During evaporation, the pressure was reduced from 250 mbar for the first 30 min to 70 mbar for the final 10 min of a run, which was sufficient to evaporate all hexane.

The pellet that remained in the 15 ml tubes was suspended again in 7.5 ml hexane for the second round of hexane extraction. After the second hexane extraction, the hexane-pellet mixture was again centrifuged and the supernatant was added to the glass tube with extracted seed oil used in the first cycle, and the hexane was evaporated again. This procedure was repeated for the third and last round of hexane extraction. After this, the amount of oil extracted was determined as the difference of the weight of the glass tube with oil and the empty glass tube. In 10 test runs, also a fourth round of hexane extraction was carried out, but the increase in the amount of oil extracted from round 3 to round 4 was less than 2% of the total amount of oil extracted after 4 rounds of hexane extraction. The whole procedure was carried out at room temperature (20°C).

#### Fatty acid composition

The fatty acid composition of the extracted oil was determined according to a standard fatty acid methyl-ester analysis (AOCS Ce 1 h-05), after saponification and methylation of the oil with a mixture of KOH and methanol. Fatty acids were identified on the basis of the retention time of standards. The fatty acids C16:0, C16:1, C18:0, C18:1n-9, C18:2n-6 and C18:3n-3 were quantified using an Agilent GLC. The fatty acid composition was expressed in terms of the relative peak areas of these fatty acids.

#### Seedling growth and morphology

We assessed the variation between the accessions in the JEP collection for early growth and morphological traits in the seedling stage on the basis of a greenhouse experiment in Wageningen, The Netherlands. For this analysis, we selected 3 healthy seeds of each of the 182 accessions. Empty or damaged seeds or seeds showing signs of infection by fungi were discarded. Seeds were sown in 1.4 L pots on December 23, 2008 in a greenhouse nursery with a constant temperature of 20 ° at night and 25°C during the day, with a 12 hours light period. Light was provided by SON-T lamps in addition to the natural light entering in the greenhouse, reaching levels around 3 MJ m^−2^ d^−1^ (PAR, between 400 and 700 nm), equivalent to about 35 W m^−2^. Germination took place approximately 14 days after sowing, but was variable between accessions. To allow proper comparison of the accessions, the timing of all plant measurements were expressed in days after germination (DAG) instead of days after sowing. After germination, individual plants were transferred to individual pots of 1.4 L and to a larger greenhouse, also with 12 hours of artificial light and a constant temperature of 20°C (night) and 25°C (day), where the plants were put according to a randomized block design with 3 blocks with one replicate plant per accession per block. Some accessions only occurred in two blocks, if one of the three replicate seeds had not germinated. Plants received a slow release NPK-fertilizer at the start of the experiment to avoid growth limitations due to nutrient shortage and plant were watered once every three days to prevent growth limitations due to water shortage.

The time until germination, until appearance of the cotyledons and the number of cotyledons were determined within 2 to 4 weeks after planting. Other non-destructive measurements of morphological traits were obtained at 30 and 60 days after germination (Table 9). Sixty days after germination, plants were fully harvested and fresh and dry weight (after drying 24 h in a stove at 105°C) of root, stem, petiole and leaves was measured, and total leaf area per plant was determined (Table 10). From total leaf area and leaf weight, the specific leaf area (SLA, cm^2^ g^−1^) was calculated. Further, dry matter distribution was determined by calculating the percentage of leaves (LWR), petioles (PWR), stems (SWR) and root (RWR) in the total biomass (Table 10). The phyllochron, i.e. the time between the appearances of successive leaves on the stem was derived from the observed number of leaves on successive points in time. The radiation use efficiency was derived from the measured photosynthetically active radiation (PAR) and the light interception estimated from the plant leaf area at final harvest. Table 11 lists these growth and morphology plant variables. In addition, for each accession, the average and variance of seed weight was determined on a sample of twenty seeds.

**Table 9:** Non-destructive plant variables measured in seedlings under greenhouse conditions

**Trait**	**Abbreviation**	**Date of measurement**	**Description**
Days to germination (d)	D_G	2-4 weeks after sowing	From day of sowing, number of days until appearance of shoot from soil.
Days to cotyledon emergence (d)	D_CE	2-4 weeks after sowing	From day of sowing, number of days until both cotyledons were fully open.
Number of cotyledons (#)	N_Cot	2-4 weeks after sowing	Number of cotyledons per plant.
Plant height (cm.)	P_H	30 DAG	Plant height from the plant base at the soil to its apex.
Stem diameter (cm.)	S_D	30 DAG	Stem diameter at the base of the trunk.
First leaf length (cm.)	FL_l	30 DAG	For the first emerged leaf, length was measured from the point at which the petiole joins the leaf, to the tip of the leaf.
First leaf width (cm.)	FL_W	30 DAG	For the first emerged leaf, width was measured between the two furthest horizontal (and parallel) points across the leaf.
Total number of leaves (#)	Nr_L	60 DAG	Overall number of mature leaves, excluding cotyledons and emerging leaves around the plant’s apex.

Sowing date was December 20, 2008. DAG = days after germination.

**Table 10:** Plant variables measured in seedlings under greenhouse conditions determined at destructive harvest of plants on day 60 after germination

**Trait**	**Abbreviation**	**Description**
Total leaf area (cm^2^)	TLA	Surface area for every mature leaf of the plant, including cotyledons but excluding emerging leaves around the plant’s apex, was measured using a Li-COR Model 3100 Area Meter (Li-COR). Total leaf area was calculated as the cumulative sum of the surface area of all leaves.
Petiole fresh and dry weight (g plant^−1^)	P_FW/P_DW	Petioles were cut off from the plant and weighed fresh and after drying at 105°C for 48 hours.
Leaf fresh and dry weight (g plant^−1^)	L_FW/L_DW	Leaves, excluding petioles) were cut off from the plant and weighed fresh and after drying at 105°C for 48 hours.
Stem fresh and dry weight (g plant^−1^)	S_FW/S_DW	The stem cut off from the plant and weighed fresh and after drying at 105°C for 48 hours.
Root fresh and dry weight (g plant^−1^)	R_FW/R_DW	Roots were cut off from the plant and weighed fresh and after drying at 105°C for 48 hours.

**Table 11:** J. curcas derived growth variables in seedlings under greenhouse conditions on day 60 after germination

**Trait**		**Description / Calculation**
Total fresh weight (g plant^−1^)	T_FW	Σ (P_FW, L_FW, S_FW, R_FW)
Total dry weight (g plant^−1^)	T_DW	Σ (P_DW, L_DW, S_DW, R_DW)
Shoot/root ratio (−)	S/R	Σ (P_DW, L_DW, S_DW)/R_DW
Leaves/stem ratio (−)	L/S	Σ (P_DW, L_DW)/S_DW
Petiole/leaf sheath ratio (−)	P/L_Sth	P_DW/L_DW
Leaf area average (cm^2^ plant^−1^)	L_AA	TLA/Σ (Nr_L, N_Cot)
Specific leaf weight (g cm^−2^)	SLW	L_DW ∙10^−3^/TLA ∙10^−8^
Specific leaf area (cm^2^ g^−1^)	SLA	TLA ∙10^−4^/L_DW
Phyllochron in days (d)	Phy_D	(Number of Days from Emergence Date till Harvest Date)/Nr_L
Phyllochron in temperature (°C)	Phy_T	(Cumulative temperature between Cotyledon Emergence Date and Harvest Date)/Nr_L
Absolute growth rate (g d^−1^)	AGR	T_DW/(Number of Days from Emergence Date till Harvest Date)
Temperature sum per DW (°C g^−1^)	TS_DW	(Cumulative temperature between Emergence Date and Harvest Date)/T_DW
Radiation use efficiency (MJ_int_ g^−1^)	RUE2	PAR Intercepted/T_DW

In the period just after the establishment of seedlings, plant growth is approximately exponential, which means that the plant will grow with a constant relative growth rate (RGR). The absolute daily growth rate is equal to product of the relative growth rate and the plant weight. RGR is calculated from growth data as (Eq. 1):

(1) $$\mathrm{RGR}=\ln\left(W_{2}/W_{1}\right)/\left(t_{2}-t_{1}\right)$$

In which, RGR is the relative growth rate (d^−1^), W_1_ and W_2_ are the plant total dry weights (g plant^−1^) at time t_1_ and t_2_ (in days after germination).

RGR can be analysed as the product of three components as (Eq. 2):

(2) RGR=SLALWRNAR

In which, SLA is the Specific Leaf Area, which is the leaf area divided by the leaf dry weight, LWR is the Leaf Weight Ratio, which is the dry weight of leaves divided by the total plant dry weight (expressed as percentage) and NAR is the Net Assimilation Rate, which in the growth analysis is calculated from the measured RGR, SLA and LWR [40].

### Data analysis of seed and seedling traits

As some accessions were only present in two blocks, residual maximum likelihood (REML) procedure was used to test significance of difference between accessions, with blocks and rows within block as random term. It proved that the row within block variance was negligible, and therefore only results using the REML procedure with blocks as random term and accessions as fixed term are presented. Statistical significance was based on a probability of 5% of falsely rejecting the hypothesis that no differences were present using the Wald statistic.

From the REML analysis, estimates of genetic variance (the variance among accessions) and the residual variance were obtained, and used to calculate the broad sense heritability h^2^ (or repeatability) of the measured traits for entry means as (Eq. 3):

(3) $$h^{2}=\sigma_{g}^{2}/\left(\sigma_{g}^{2}+\left(\frac{\sigma_{e}^{2}}{3}\right)\right)$$

Where $\sigma_{g}^{2}$ is the variance across all accessions and $\sigma_{e}^{2}$ is the residual variance of the experiment. The coefficient of genetic variation was calculated as $\sigma_{g}^{2}$/mean.

Differences in means of different geographical origins of the accessions were tested using a REML analysis with region as fixed term instead of accessions. To test whether the (genetic) variance among accessions from different regions were significantly different, pairwise F-tests on the ratio of variance of one region and that of another region were performed.

We carried out a principal component analysis (PCA) analysis using the correlations between seedling growth and morphological traits to assess whether the total set of traits would show a structuring into groups of several accessions with high similarity (using Genstat 11^th^). An alternative presentation in the form of a dendrogram showing possible structuring into group of high similarity was based on Euclidean distances calculated from standardized values for the traits measured.

## Competing interests

Financial competing interests

In the past five years have you received reimbursements, fees, funding, or salary from an organization that may in any way gain or lose financially from the publication of this manuscript, either now or in the future? Is such an organization financing this manuscript (including the article-processing charge)? NO.

Do you hold any stocks or shares in an organization that may in any way gain or lose financially from the publication of this manuscript, either now or in the future? If so, please specify? NO.

Do you hold or are you currently applying for any patents relating to the content of the manuscript? Have you received reimbursements, fees, funding, or salary from an organization that holds or has applied for patents relating to the content of the manuscript? If so, please specify. NO.

Do you have any other financial competing interests? If so, please specify. NO.

Non-financial competing interests

Are there any non-financial competing interests (political, personal, religious, ideological, academic, intellectual, commercial or any other) to declare in relation to this manuscript? If so, please specify. NO.

## Authors’ contributions

LRM, AT, REEJ, ENvL, CA, LMT, RGFV: Marker selection and designed experiments. LRM, AT, JB: developed molecular markers. LRM, AT, REEJ, ENvL: phenotypic evaluation. LRM, AT, ENvL: biodiversity analysis (molecular marker and phenotypic data). LRM, REEJ, LMT, RGFV, ENvL prepared the manuscript. All authors: reviewed and approved manuscript.
